# Comparative Proteomic Analyses of Avirulent, Virulent, and Clinical Strains of *Mycobacterium tuberculosis* Identify Strain-specific Patterns*[Fn FN2]

**DOI:** 10.1074/jbc.M115.666123

**Published:** 2016-05-05

**Authors:** Gagan Deep Jhingan, Sangeeta Kumari, Shilpa V. Jamwal, Haroon Kalam, Divya Arora, Neharika Jain, Lakshmi Krishna Kumaar, Areejit Samal, Kanury V. S. Rao, Dhiraj Kumar, Vinay Kumar Nandicoori

**Affiliations:** From the ‡ National Institute of Immunology and; the ¶ Cellular Immunology Group, International Centre for Genetic Engineering and Biotechnology, Aruna Asaf Ali Marg, New Delhi 110067,; the § Drug Discovery Research Center, Translational Health Science and Technology Institute, Faridabad, Haryana 121004, and; the ‖ Institute of Mathematical Sciences, Chennai 600113, India

**Keywords:** mycobacteria, Mycobacterium tuberculosis, protein expression, proteomics, virulence factor

## Abstract

*Mycobacterium tuberculosis* is an adaptable intracellular pathogen, existing in both dormant as well as active disease-causing states. Here, we report systematic proteomic analyses of four strains, H37Ra, H37Rv, and clinical isolates BND and JAL, to determine the differences in protein expression patterns that contribute to their virulence and drug resistance. Resolution of lysates of the four strains by liquid chromatography, coupled to mass spectrometry analysis, identified a total of 2161 protein groups covering ∼54% of the predicted *M. tuberculosis* proteome. Label-free quantification analysis of the data revealed 257 differentially expressed protein groups. The differentially expressed protein groups could be classified into seven K-means cluster bins, which broadly delineated strain-specific variations. Analysis of the data for possible mechanisms responsible for drug resistance phenotype of JAL suggested that it could be due to a combination of overexpression of proteins implicated in drug resistance and the other factors. Expression pattern analyses of transcription factors and their downstream targets demonstrated substantial differential modulation in JAL, suggesting a complex regulatory mechanism. Results showed distinct variations in the protein expression patterns of *Esx* and *mce1* operon proteins in JAL and BND strains, respectively. Abrogating higher levels of ESAT6, an important Esx protein known to be critical for virulence, in the JAL strain diminished its virulence, although it had marginal impact on the other strains. Taken together, this study reveals that strain-specific variations in protein expression patterns have a meaningful impact on the biology of the pathogen.

## Introduction

The ability of a pathogen to survive under harsh conditions within the host is connected to its ability to modulate host cellular processes to its advantage. The advent of multiple drug-resistant and extensive drug-resistant *Mycobacterium tuberculosis* strains is a major global heath concern, compromising the existing therapy (1–3). Despite the best efforts, the mechanisms underlying the pathogenesis, virulence, and persistence of *M. tuberculosis* infection associated with the drug-resistant strains is not very well understood. The identification of virulence factors that are required for disease progression is critical for understanding the biology of infection.

The availability of whole genome sequences of different *M. tuberculosis* strains (4–6) has enabled genome-wide comparisons to identify the presence of deletions or gene mutations that correlate with virulence (7). In recent years systems biology approaches, which study complex interactions, have been successfully applied to predict the networks and dynamic interactions between pathogen and host (8, 9). Various genome-wide studies comparing drug-sensitive and drug-resistant *Mycobacterium* strains have identified multiple single nucleotide polymorphisms related to DNA repair, replication, and recombination genes, thereby providing insights into the genetic basis of drug resistance (10–13). Transcriptomics analyses of multiple drug-resistant strains in comparison with drug-sensitive strains have highlighted the role of altered gene expression of type II fatty-acid synthases, efflux genes, central metabolic pathway members, ABC transporters, and genes related to stress response (14–17).

Quantitative protein expression profiling has proven to be a useful method in understanding how mycobacterial species adapt to different stress conditions. Previous studies have utilized differential growth conditions *in vitro* to mimic stress conditions and have identified cellular markers for these stress conditions with the help of two-dimensional gel electrophoresis-based approaches (18–21). Two-dimensional gel electrophoresis-based approaches have also been used to identify strain-specific differences among virulent and avirulent strains of *M. tuberculosis* (18, 19, 22, 23). With advances in technology, differential proteomic analysis has emerged as a valuable tool in generating large datasets to elucidate complex biological systems (24–26). Quantitative proteomics studies have highlighted differential expression of proteins among *M. tuberculosis* strain H37Rv and *Mycobacterium bovis* BCG, particularly in relation to lipid biosynthesis pathways (18, 27) as well as during different phases of growth and nutrient starvation (28, 30). In a related study, quantitative proteomic analysis with the help of dimethyl labeling was utilized to investigate the carbon assimilation process in *Mycobacterium smegmatis* (31). Using a combination of discovery and targeted approaches, a selected reaction monitoring-based *M. tuberculosis* proteome library was recently generated to accurately quantitate the proteins of *M. tuberculosis* and related clinical strains (32). Despite several reports regarding the *M. tuberculosis* proteome, very few proteomic studies have been performed on drug-resistant clinical strains (33).

Although efforts have been focused on identifying secreted as well as intracellular mycobacterial proteins, less attention has been paid toward comparing the protein profiles of clinical isolates with commonly used laboratory-adapted strains such as H37Rv. This report details the results of a systematic whole cell proteome analysis of the laboratory avirulent strain H37Ra, laboratory virulent strain H37Rv, single drug-resistant clinical isolate BND-433, and multidrug-resistant clinical isolate JAL-2287.

## Materials and Methods

### 

#### 

##### Bacterial Growth Conditions

All the bacterial strains (H37Rv (Rv), H37Ra (Ra), BND-433 (BND), and JAL-2287 (JAL)) used in the study were grown in Middlebrook 7H9 media (Difco) supplemented with 0.2% (v/v) glycerol, 10% albumin dextrose/catalase, and 0.05% Tween 80 at 37 °C. Cells were harvested at middle-late log phase (*A*_600_ ∼1–1.5), and bacterial pellets were washed twice with TBST (20 mm Tris-HCl, pH 7.5, 150 mm NaCl, 0.1% Tween 20) and once with TBS (20 mm Tris-HCl, pH 7.5, 150 mm NaCl) and stored at −80 °C.

To monitor the growth of mycobacteria in different growth media, 10 × 10^6^ bacteria/ml for each strain were washed with PBS (20 mm phosphate buffer, pH 7.4, 150 mm NaCl) and added to two different carbon source (0.05% oleic acid or 0.05% cholesterol)-supplemented 7H9 growth media formulations. Alamar Blue (0.01%) (Life Technologies, Inc.) was added to these cultures, and the reduction of Alamar Blue was monitored from 0 to 30 h by measuring the absorbance according to the manufacturer's instructions.

##### Sample Preparation

Cell pellets were resuspended in lysis buffer (8 m urea in 25 mm ammonium bicarbonate) supplemented with complete protease and phosphatase inhibitor mixture (Roche Applied Science). The cells were lysed within a bead beater with the help of 0.1-mm zirconium beads. The cells were disrupted in a Mini bead-beater for 10–12 cycles (45-s pulse with 60-s incubation). Lysates were clarified by centrifugation, and the concentrations were determined by the Bradford assay (Bio-Rad).

50 μg of the sample was first reduced with 5 mm tris(2-carboxyethyl)phosphine and further alkylated with 50 mm iodoacetamide. The samples were diluted to 1 m final urea concentration with 25 mm ammonium bicarbonate buffer and digested with trypsin (1:50, trypsin/lysate ratio) for 16 h at 37 °C. Digests were cleaned using a C18 silica cartridge (The Nest Group, Southborough, MA) according to the manufacturer's protocol and dried using a speed vac. The dried pellet was resuspended in buffer A (5% acetonitrile, 0.1% formic acid).

##### Mass Spectrometric Analysis of Peptide Mixtures

All the experiments were performed using EASY-nLC system (Thermo Fisher Scientific) coupled to LTQ Orbitrap-Velos mass spectrometer (Thermo Fisher Scientific) equipped with nanoelectrospray ion source. 1 μg of the peptide mixture was resolved using a 10-cm PicoFrit Self-Pack microcapillary column (360-μm outer diameter, 75-μm inner diameter, 10-μm tip) filled with 5 μm of C18-resin (Magic). The peptides were loaded with buffer A and eluted with a 0–40% gradient of buffer B (95% acetonitrile, 0.1% formic acid) at a flow rate of 300 nl/min for 120 min. This was followed by a 10-min gradient of 40–80%, 20-min gradient of 80–90%, and finally equilibrated with buffer A for 30 min.

The LTQ Orbitrap-Velos was operated using the Top10 higher energy collisional dissociation (High/High) data-dependent acquisition mode (34) with a full scan in the Orbitrap and an MS/MS scan in the higher energy collisional dissociation. The target values for the full scan MS spectra were set at 1 × 10^6^ charges with a maximum injection time of 200 ms, and a resolution of 30,000 at *m*/*z* 400. MS/MS scans were acquired at a resolution of 7500 at *m*/*z* 400 with an ion target value of 1 × 10^4^ with a maximum injection time of 200 ms. Lock mass option was enabled for polydimethylcyclosiloxane ions (*m*/*z* = 445.120025) for internal recalibration during the run.

##### Data Processing

Four biological replicates were processed for each strain, and the 16 RAW files generated were analyzed with MaxQuant (version. 1.4.1.2) against the *M. tuberculosis* UniProt reference proteome database. For Andromeda search, the precursor and fragment mass tolerances were set at 10 ppm and 0.5 Da, respectively. The protease used to generate peptides, *i.e.* enzyme specificity was set for trypsin/P (cleavage at the C terminus of “K/R: unless followed by “P”) along with maximum missed cleavages value of two. Carbamidomethyl on cysteine as fixed modification and oxidation of methionine and N-terminal acetylation were considered as variable modifications for database search. The settings also included reverse sequences of database and in-built 247 contaminants in default settings of MaxQuant. For peptide identification, a peptide posterior error probability threshold of 0.05 was specified. Both peptide spectrum match and protein false discovery rate were set to 0.01. Default settings were applied for all other parameters.

For quantitative comparison among the biological replicates of the four strains, label-free quantification with “match between runs” option was utilized within the MaxQuant suite with standard settings for generating the peak lists from 16 raw files (Xcalibur and ThermoFisher Scientific) with a retention time alignment window of 1 min. Protein groups were created by default settings of MaxQuant in case the peptide sets were common among multiple proteins. For dynamic range estimation, iBAQ^3^ analysis was also performed with default settings.

##### Bioinformatics Analysis

Bioinformatics analysis was performed using Perseus software. UniProt annotation for *M. tuberculosis* reference proteome database was utilized for GO classification of all the identified proteins. Custom-made perl scripts were utilized for correlating *Mycobacterium* UniProt identifiers with tuberculist database protein entries. *Z*-score normalization was performed on the log2 transformed LFQ values obtained from label-free quantification analysis. The 16 samples were divided into four strain groups, and ANOVA test was performed with Benjamini Hochberg correction. ANOVA-significant proteins were utilized for hierarchical clustering analysis. Principal component analysis (PCA) was performed using ggbiplot package in R (3.1.0), whereas K-means clustering was performed using the Past analysis tool. Different graphics were generated using a combination of R (3.1.0 version), Graphpad, and Excel software. The mass spectrometry proteomics data have been deposited to the ProteomeXchange Consortium (35) via the PRIDE partner repository with the dataset identifier PXD001188.

##### ESAT-6 Antibody Treatment

10 × 10^6^ bacteria for each mycobacterial strain were washed with PBS and either treated with ESAT-6 antibody (ab26246) or not treated for 45 min at room temperature. Untreated and ESAT-6 antibody-treated cultures were processed for infection of PMA-differentiated THP-1 cells at a multiplicity of infection of 10. Infection was maintained at 37 °C in a CO_2_ incubator for 4 h followed by 2 h of amikacin treatment to remove any extracellular bacteria. Infected cells were washed with RPMI 1640 medium followed by lysis using 0.06% SDS and plated on 7H11 agar plates to determine the CFUs. 15 days post-plating CFUs for each strain were counted and averaged between multiple sets.

## Results

### 

#### 

##### Proteome Analysis of Virulent and Avirulent M. tuberculosis Strains

**FIGURE 1.**
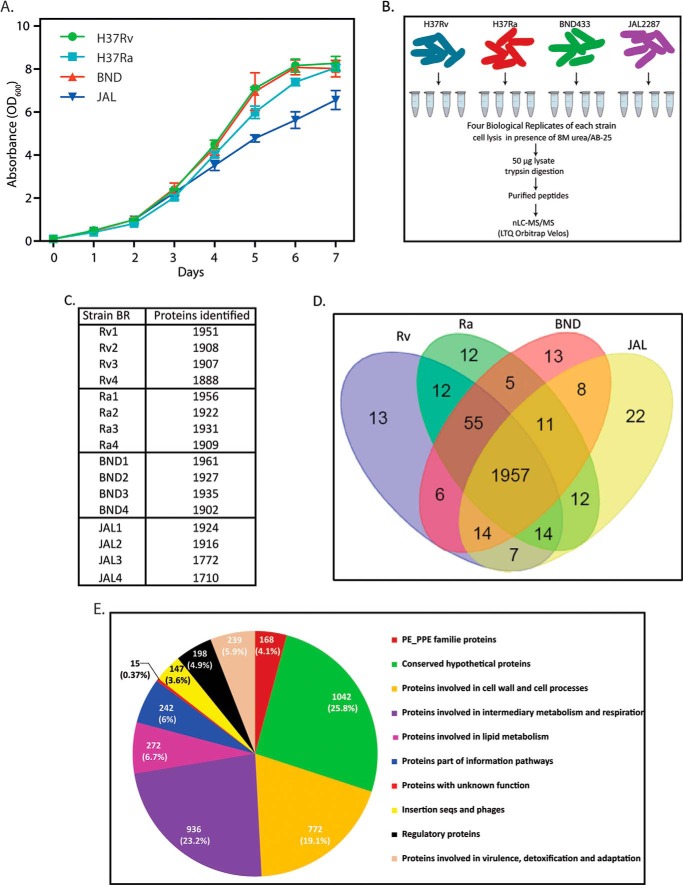
**Proteome analysis of virulent and avirulent *M. tuberculosis* strains.** *A, in vitro* growth analysis of mycobacterial strains H37Ra, H37Rv, BND, and JAL in 7H9 medium. All the cultures were seeded at an initial *A*_600_ of 0.05, and growth was monitored every 24 h for 7 days. *B,* schematic illustration of the design and workflow to perform the quantitative proteomics analysis across the four *Mycobacterium* strains. Cell pellets were lysed with 8 m urea/AB-25, and equal amounts of lysates were trypsin-digested, and the purified peptides were identified using high resolution LC-MS/MS analysis using LTQ Orbitrap Velos instrument. *C,* raw MS files were processed with MaxQuant (version. 1.4.1.2) against the *M. tuberculosis* UniProt reference proteome database. The false discovery rate was set at 0.01 for both peptides and proteins. Bioinformatics analysis was performed using Perseus software. Custom-made perl scripts were utilized for correlating *Mycobacterium* UniProt nomenclature with tuberculist database entries. The *table* shows the number of proteins identified for each biological replicate for all four strains. *D,* the *Venn diagram* shows the number of unique and common proteins among the four strains. *E, pie chart* shows the details of tuberculist classification of functional categories of *M. tuberculosis* proteome.

BND-433 and JAL-2287 strains are CAS clade clinical strains that are resistant either to only streptomycin or streptomycin, isoniazid, and rifampicin, respectively (36). Prior to proteomic analysis of the four strains, we monitored their growth profiles in 7H9 medium every 24 h over 8 days. The growth profiles for all four strains were found to be similar until day 5 (Fig. 1*A*). To perform comparative proteomics analysis, cells were harvested on day 3, and four biological replicates for each strain were processed using the proteomic workflow shown in Fig. 1*B*. The raw files were searched against Uniprot *M. tuberculosis* reference proteome database, and a total of 2161 protein groups were identified with high confidence using 1% protein and peptide false discovery rate cutoff (supplemental Tables 1–3). The identified proteins covered ∼54% of the predicted *M. tuberculosis* proteome, with each run identifying between 1700 and 1900 protein groups (Fig. 1*C*). Comparative analysis of the protein groups of Rv, Ra, BND, and JAL strains revealed 13, 12, 13, and 22 strain-specific protein groups, respectively (Fig. 1*D*). Among them 3, 2, 3, and 18 protein groups were detected in at least two biological replicates of the particular strain, respectively, and not in the others (supplemental Table 4).

To categorize proteins according to their functional relevance, we first organized the *M. tuberculosis* database proteins (a total of 4031 proteins) into functional categories as per the tuberculist classification (Fig. 1*E*). The highest numbers of proteins were conserved hypotheticals, cell wall and cell process-related, and those involved in intermediary metabolism and respiration (Fig. 1*E*). The 2161 proteins identified in the proteome as part of this study were distributed over all the functional categories, with the majority of the proteins belonging to the categories of intermediary metabolism and respiration (31%), conserved hypotheticals (22.2%), and cell wall and cell process-related (17.2%), constituting ∼66% of the total identified proteome.

##### Relative Protein Quantification

**FIGURE 2.**
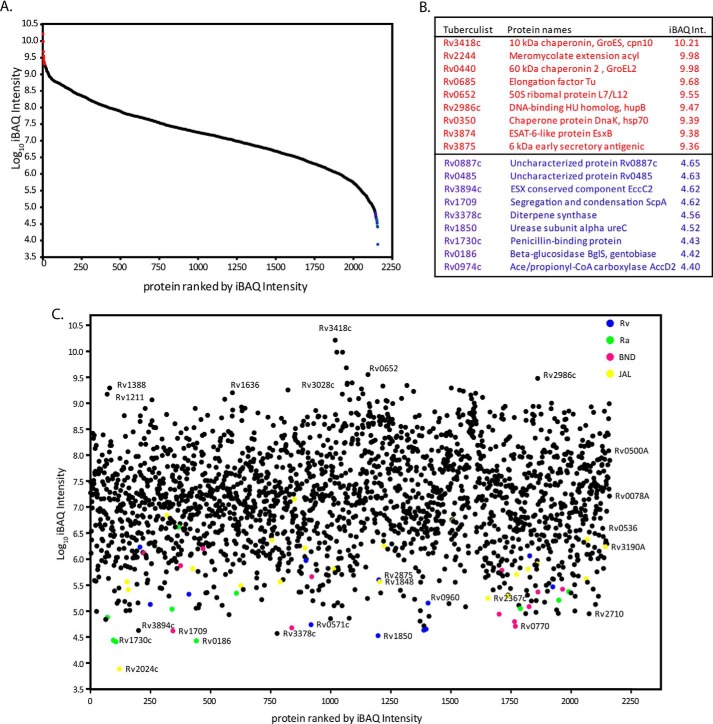
**iBAQ analysis and dynamic range estimation of identified mycobacterium proteome.** *A,* combined iBAQ protein expression values for the 2157 proteins were plotted with log_10_iBAQ intensity on the *y* axis, and proteins were ranked by iBAQ intensity on the *x* axis. Plot reveals a dynamic range of 6 orders of magnitude (*left panel*). *B,* list of nine most (colored *red*) and least (colored *blue*) abundant proteins based on iBAQ intensity. *C,* strain-specific protein expression plot based on iBAQ intensities. Rv, Ra, BND, and JAL strain-specific proteins were represented in *blue, green, pink,* and *yellow*.

To estimate the abundance of identified proteins, iBAQ was carried out using MaxQuant software package. This method takes into consideration the summation and normalization of MS signals based on peptide size, length, and number of theoretical peptides possible for all the proteins identified in a particular proteome run (26, 37). The iBAQ intensity in the composite proteome spanned a dynamic range of 6 orders of magnitude between the most and least abundant proteins (Fig. 2 and supplemental Table 5). The most abundant proteins thus identified were chaperones, elongation factors, ribosomal proteins, chromosomal architectural proteins, and secretory proteins. Many of these protein families, such as heat shock proteins (including chaperones), ribosomal proteins, and ribosomal translational machinery, are highly expressed in several organisms, including mycobacterial species (37, 38). Plotting all the identified proteins on the iBAQ protein abundance scale indicated that the majority of strain-specific proteins (supplemental Table 5) were present in the low abundance range. However, some JAL-specific proteins were found in the moderate abundance range.

**FIGURE 3.**
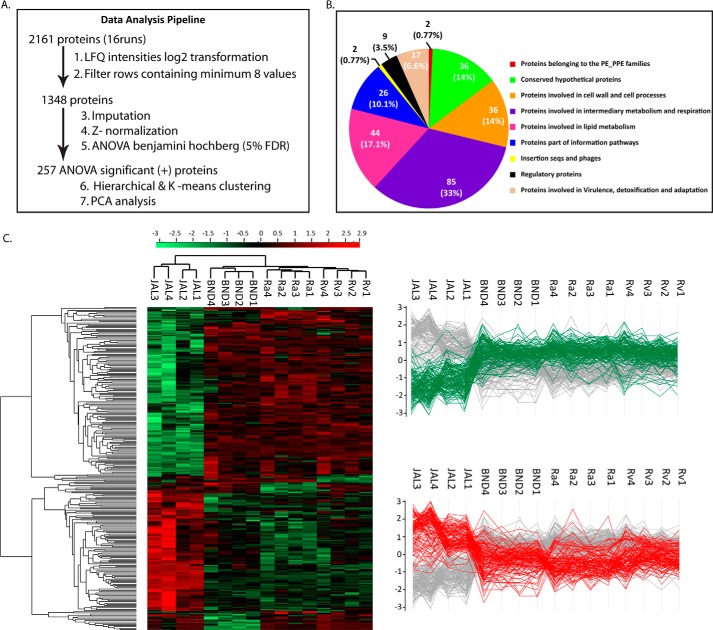
**Relative protein quantification.** *A,* brief overview of data analysis steps. *B, Venn diagram* showing details of tuberculist classification of functional categories among the ANOVA-significant proteins. *C, left panel,* clustergram of the ANOVA-significant 257 proteins differing among the four strains. The *Z*-scored normalized abundance values of proteins are represented by *red* (high abundance) and *green* (low abundance) colors as indicated in the *color scale bar* at the top. The two highlighted clusters represent proteins with decreased (*green*) and increased (*red*) abundance in the JAL strain. *Right panel,* profile plots displaying the low and high abundant clusters of clustergram with respect to the JAL strain.

LFQ methodology was used to compare the levels of identified proteins among the four strains (39). To cross-compare the reproducibility of results across the biological replicates, a correlation matrix was generated (data not shown). The Pearson correlation of 0.8–0.9 among the biological replicates suggested low variance and high reproducibility, and these multiple scatterplots also showed a larger spread of LFQ values in the inter-strain comparison. Among the 2161 proteins identified in the composite proteome, we were able to quantify 1348 proteins after application of LFQ (Fig. 3*A*; quantification values from 16 samples; minimum row filter ≥8). Further box plots generated after *Z*-score median normalization of these 1348 proteins also showed high reproducibility among the biological replicates (data not shown).

##### Proteome of the JAL Strain Is Significantly Different from Ra, Rv, and BND

To investigate differential expression among the four strains, we applied Benjamini-Hochberg correction for multiple hypotheses testing using one-way ANOVA test with a cutoff value of 0.05 (Fig. 3*A*). Among the 257 differentially modulated proteins (supplemental Table 6), a majority of the proteins belonged to categories of intermediary metabolism and respiration (33%), lipid metabolism (17.1%), cell wall and cell process-related (14%), and conserved hypotheticals (14%), with ∼10% belonging to the category of information processing pathways (Fig. 3*B*). The supplemental Table 7, *A* and *B* shows a summary of statistical variations among the biological replicates in the 257 ANOVA significant protein groups. These 257 protein groups were further subjected to unsupervised hierarchical clustering, and the resulting clustergram is shown in Fig. 3*C* (*left panel*). Scrutiny of the clustergram indicated that the large numbers of proteins are either up- or down-regulated in the JAL strain when compared with the remaining three strains. The two major clusters of proteins displaying prominent up- and down-regulation in the JAL strain were further arranged as profile plots (Fig. 3*C, right panel*).

**FIGURE 4.**
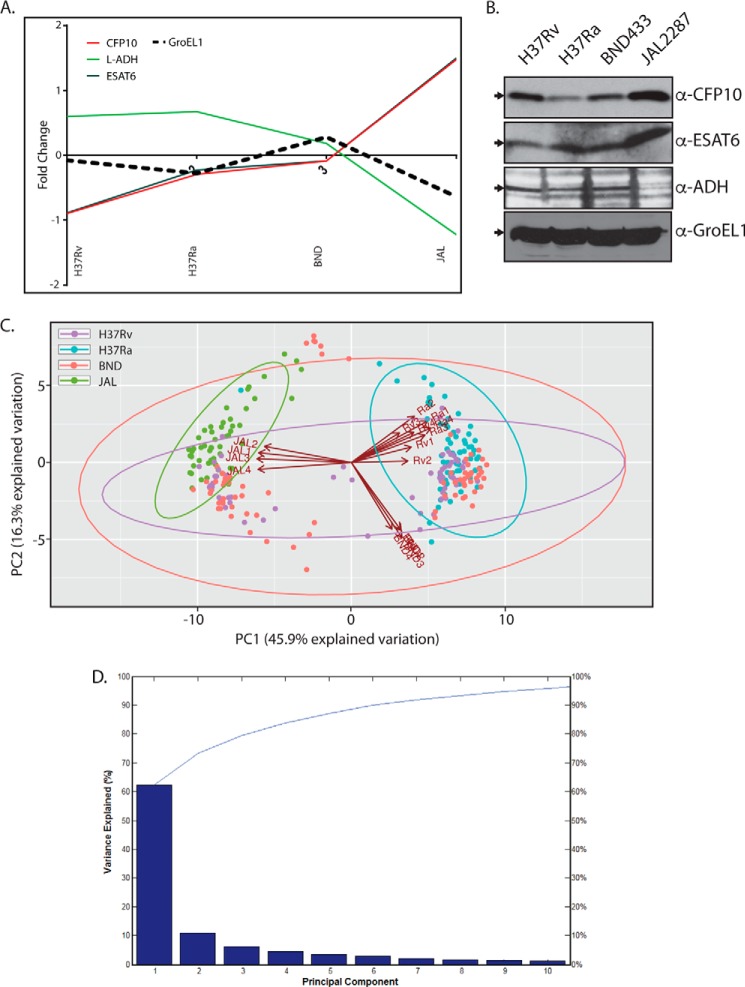
**PCA analysis and validation of significant proteins.** *A,* profile plots showing the fold change analysis of selected genes, which were up- or down-regulated in the JAL strain. The median values obtained for each strain were plotted on the *y* axis. *B,* whole cell lysates were estimated with the help of the BCA assay kit (Thermo). 50 μg of whole cell lysates were resolved on SDS-PAGE, transferred to nitrocellulose membrane, and probed separately with α-CFP10, α-ESAT6, and α-ADH antibodies (Abcam). 7.5 μg of whole cell lysates were used for probing with α-GroEL1 antibody (control). *C,* biplots of a principal component analysis performed on mycobacterial cell lines measured in quadruplicate. Matlab was utilized for PCA. The *arrows* indicate the loadings of the cell lines (Rv, Ra, BND, and JAL). All 257 ANOVA significant proteins were used in the analysis. *D,* Parreto's plot for the principal components (Fig. 3*C*). Fractions of variability in the data captured by each of the principal components are shown here as *bars*. The cumulative variance captured by the principal components is represented as the *line*. Note that first two principal components together account for more than 70% of the variance.

To validate the results obtained using the above workflow, we selected CFP-10, ESAT-6, l-ADH, and GroEL1 as test proteins in Western blotting analyses of whole cell lysates. Although l-ADH (belonging to the category of intermediary metabolism and respiration) is down-regulated in JAL, the secretory protein CFP-10 and ESAT6 were up-regulated in JAL (Fig. 4*A*). The *M. tuberculosis* housekeeping protein GroEL-1, a primary chaperonin, which showed minimal variations across the strains, was selected as controls. We found that although the GroEL1 expression pattern was similar across all the four strains, l-ADH expression was lower in JAL compared with the other strains, and CFP-10 and ESAT6 expressions were considerably higher (Fig. 4*B*). Thus, the expression profiles detected by Western blots were found to be reasonably consistent with the results obtained with LFQ quantification, validating our experimental approach.

In general, the JAL strain displayed substantial differential regulation of proteins as compared with the other strains (Figs. 3*C* and 4, *A* and *B*). PCA was performed on the differentially regulated proteome data sets across the strains and replicates (Fig. 4*C*). In keeping with the finding that there are significantly higher numbers of differentially regulated proteins in the JAL strain, on the principal component 2 (PC2) *versus* PC1 plot all the JAL replicates clustered diametrically opposite to the other three strains (Fig. 4*C*). The first two principal components were most significant as together they captured more than ∼62% of the variations in the data (Fig. 4*D*). Although each of the Ra and Rv replicates clustered in the same quadrant, BND replicates clustered in the adjacent quadrant (Fig. 4*C*).

We classified the proteins into strain-specific ellipses, encircling molecules with significant projections on respective principal components. Molecules showing significant projections in JAL clustered together in the PC2 *versus* PC1 plot alongside its loading vectors (Fig. 4*C*). Similarly, molecules showing significant importance in Ra clustered corresponding to its loading vectors. However, molecular vectors for Rv and BND were relatively less clustered, spreading to the quadrants enriched with JAL-specific molecular vectors (Fig. 4*C*). This was an interesting observation because the three strains other than Ra are virulent, and it appears that in keeping with this commonality they share features despite inherent variations. Interestingly, a set of proteins from the BND cluster was placed outside the BND ellipse. These belonged to the *mce1* operon, consisting of genes involved in the regulation of lipid metabolism in *M. tuberculosis* (40). This feature distinguished BND from the other three strains.

##### K-means Cluster Analysis

**FIGURE 5.**
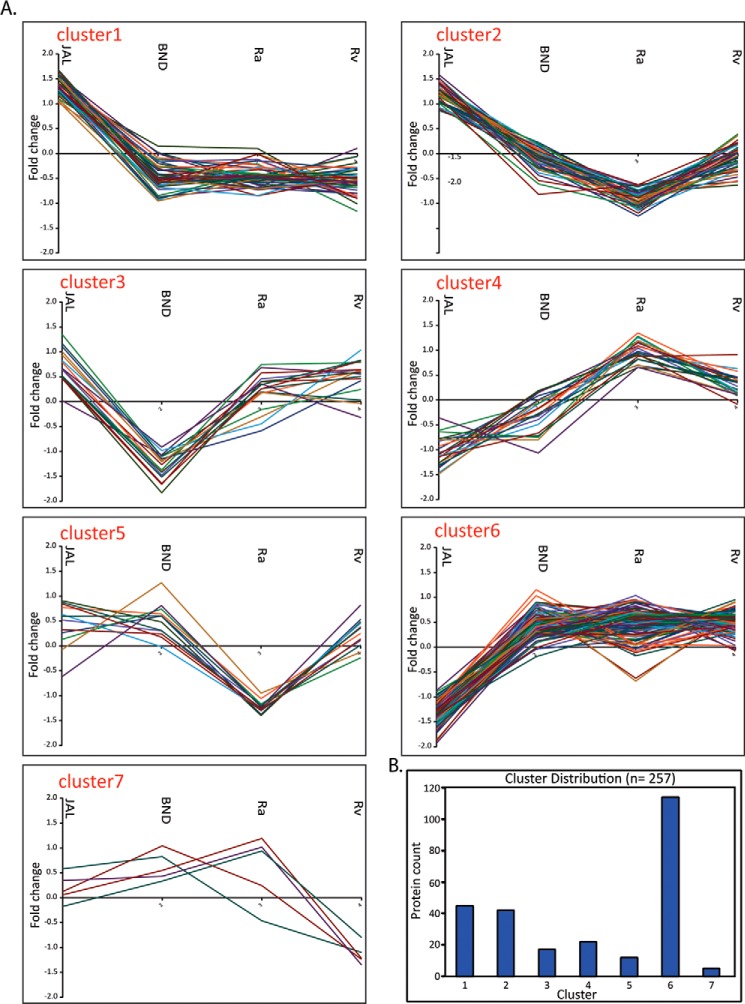
**K-means cluster analysis of ANOVA-significant proteins.** *A,* proteins showing significant protein abundance changes were grouped into seven clusters. Median of *Z*-scored log 2 normalized values for each quadruplicate was used for cluster analysis. Matlab was utilized for K-means clustering. *B, bar chart* showing the number of protein in each cluster.

We next performed K-means cluster analysis on the set of ANOVA-significant 257 proteins. K-cluster typically brings proteins having a similar expression pattern together and allows functional enrichment analysis to understand the consequences of the observed expression pattern. We decided to use seven bins for the K-cluster analysis, partly based on empirical observation of distinct clusters in the clustergram shown in Fig. 3*C*. The K-bin specific expression patterns of constituent proteins are shown in Fig. 5 and supplemental Table 8. Analysis of cluster-specific profiles revealed interesting patterns. For example, all the 45 proteins (supplemental Table 8*A*) present in cluster 1 were up-regulated in JAL and down-regulated in the other three strains (Fig. 5). Proteins in clusters 2 and 5 showed prominent down-regulation in H37Ra. Although we observed a significant up-regulation of proteins in JAL in cluster 2, in cluster 5 a comparable expression of proteins was observed in JAL, BND, and Rv. Cluster 3 contained 17 proteins, which specifically showed lower expression in BND compared with the other three strains (Fig. 5). Both clusters 4 and 6 showed down-regulation of proteins in the JAL strain. Proteins present in cluster 4 showed the highest expression in Ra and moderate expression in Rv strain, with low expression in BND and lowest in JAL strain (Fig. 5).

**FIGURE 6.**
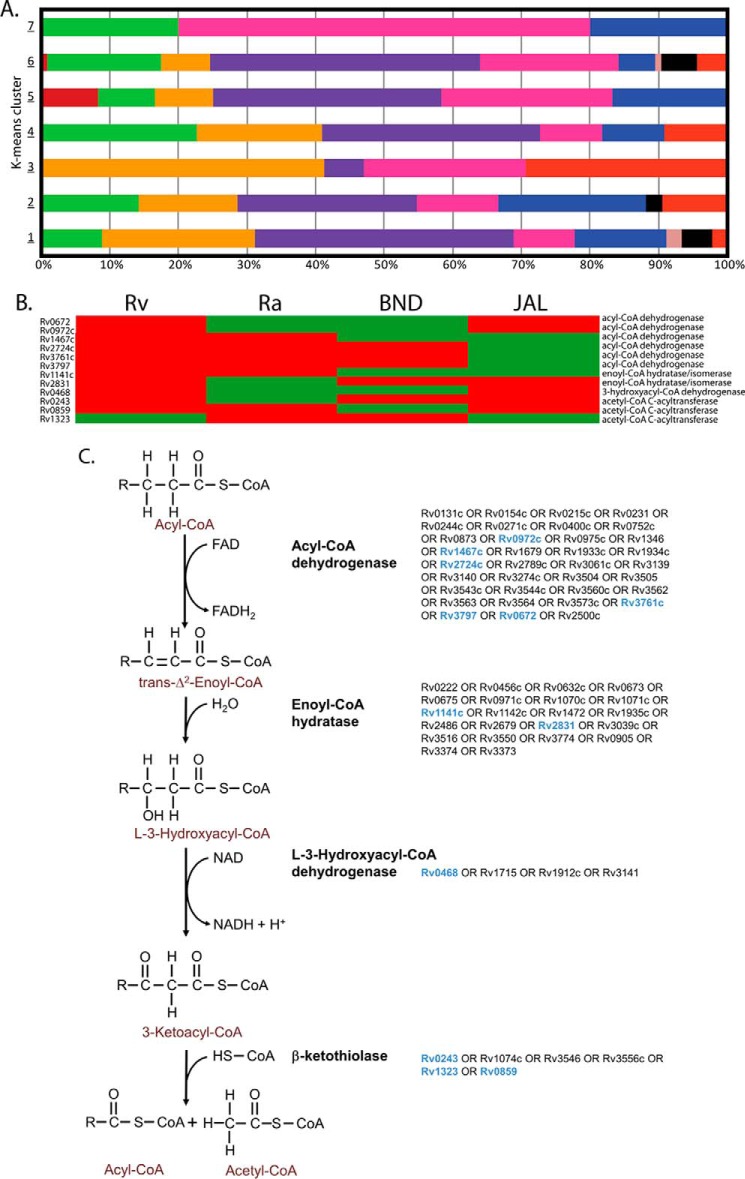
**Distribution among the K-mean clusters.** *A, bar chart* showing the percentage of tuberculist functional categories present in each cluster. *B* and *C,* metabolic pathway for the β-oxidation of fatty acids. *B,* clustergram of the 12 differentially expressed proteins across the four *M. tuberculosis* strains in the subsystem β-oxidation of fatty acids. The *Z*-scored normalized abundance values of proteins are represented by *red* (high abundance) and *green* (low abundance) colors. *C,* β-oxidation of fatty acids is accomplished by four enzyme-catalyzed reactions as given in the outline. This pathway is highly redundant at the level of genes encoding the associated enzymes for the four reactions. In the *M. tuberculosis* genome, there are 35 predicted genes for acyl-CoA dehydrogenase, 22 predicted genes for enoyl-CoA hydratase, 4 predicted genes for l-3-hydroxyacyl-CoA dehydrogenase, and 6 predicted genes for β-ketothiolase (indicated). The differentially expressed genes in the β-oxidation pathway across the four *M. tuberculosis* strains are indicated in *blue*.

Next, we addressed the question of whether strain-specific clusters were also enriched in proteins of specific functional categories. Fig. 6*A* shows classification of proteins present in each of the seven clusters into different functional classes. The percentage distribution of these functional categories is shown in Fig. 6*A*. This analysis yielded a few puzzling revelations. For example, cluster 5, whose proteins showed significant down-regulation in JAL compared with other strains, had a maximum number of genes belonging to lipid metabolism, intermediary metabolism, and hypoxia, which are considered to be critical for improved survival of pathogen in host (41). In contrast, cell wall-related proteins in clusters 1 and 2 were up-regulated in JAL. Notably, we observed that seven members of the Esx family were also part of clusters 1 and 2 (Fig. 5 and supplemental Table 8*A*). Surprisingly, we observed only 10 protein groups among the 257 that have shown similar expression patterns between JAL and BND strains (supplemental Table 8*B*). We also observed the down-regulation of the *mce1* operon proteins in cluster 3, in the single drug-resistant BND strain in comparison with other strains, suggesting a possible compromise in lipid homeostasis. Thus K-cluster analysis provided a glimpse of the strain-specific protein profile variations, which can be used to address the differential behavior of these strains in terms of virulence, survival, and drug resistance.

##### Metabolic Network Analysis

From the list of enriched functional categories across the 257 differentially expressed protein groups reported in Fig. 2*B*, it was evident that a significant fraction of differentially expressed proteins are metabolic enzymes. Thus, we decided to overlay the set of differentially expressed proteins onto the most recent genome-scale metabolic network reconstruction iOSDD890 for *M. tuberculosis* (42). We found that 90 out of the 257 differentially expressed proteins were enzymes catalyzing reactions in the metabolic model iOSDD890, and these 90 differentially expressed enzymes participate in reactions that belong to 29 different subsystems or metabolic pathways (supplemental Table 9). From the classification of the 90 differentially expressed enzymes into different subsystems of the metabolic network (supplemental Table 9), we find that 5 out of 7 differentially expressed proteins in the subsystem “membrane metabolism” and 4 out of 5 differentially expressed proteins in the subsystem “redox metabolism” are up-regulated in the JAL strain (supplemental Table 9).

Moving onto the lipid metabolism, there are 12 differentially expressed proteins across the four *M. tuberculosis* strains in the subsystem “β-oxidation of fatty acids” (supplemental Table 9 and Fig. 6*B*), of which only one protein is down-regulated in Rv strain, but six proteins were down-regulated in the JAL strain. At first glance, from the comparative analysis of the total number of down-regulated proteins in the β-oxidation pathway across strains, it may seem that this pathway for utilization of fatty acids is down-regulated in JAL compared with Rv (they all belong to cluster 6 in Fig. 5). But the β-oxidation pathway is highly redundant with several genes in the *M. tuberculosis* genome encoding each of the four enzymes catalyzing the four reactions in the subsystem (Fig. 6*B*), and any conclusions on differential activity of this pathway must be drawn in the light of the underlying redundancy in the system. On closer examination, we found that although six differentially expressed proteins in the β-oxidation pathway were down-regulated in the JAL strain, there was at least one protein associated with each of the four reactions in the pathway that was up-regulated in the JAL strain (Fig. 6*B*). Thus, by properly accounting for the redundancy in the β-oxidation pathway, we find that this pathway is not down-regulated and compromised in JAL strain.

##### Differential Modulation of Transcription Factors and Their Targets May Contribute to the Virulence of JAL

**FIGURE 7.**
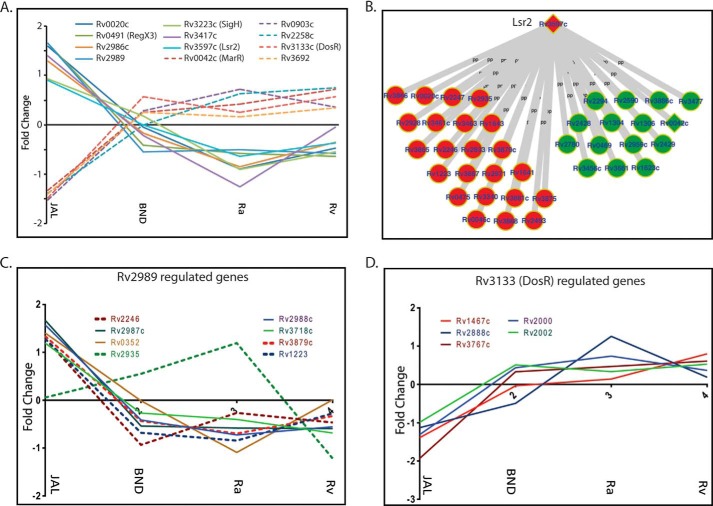
**Protein levels of transcription factors vary among the strains.** *A,* profile plots showing the fold change of different transcription factors found among the 257 ANOVA-significant proteins. *B,* Galagan network analysis of transcriptional factors. *C,* profile plots showing the fold change of the target proteins, whose expression is regulated by Lsr2. *D,* profile plots showing the fold change of the target proteins whose expression is regulated by R2839.

As the data presented in Figs. 4 and 6*A* revealed that in general the protein expression was most differentially regulated in JAL among the strains being studied here. We considered the possibility of this occurring via the enhanced/reduced expression of specific transcription factors, which would result in an increased/decreased activation of the specific target genes. Thus, we sought to investigate the expression patterns of transcription factors and their downstream targets among the ANOVA-significant proteins. Toward this, we superimposed the expression data of the 257 differentially expressed proteins on two recently reported gene regulatory networks of *M. tuberculosis* (43, 44). Analysis revealed a total of 12 of these to be transcription factors, with seven being present in both the networks. Among the 12, seven transcription factors are up-regulated and five are down-regulated specifically in the JAL strain (Fig. 7*A*). With the exception of transcription factors Rv3139c, Rv2989 and Rv3597c, we have either not found any or found only one or two targets for the remaining transcription factors among the 257 protein groups. Among the down-regulated transcription factors, the downstream targets of only one (Rv3139c/DosR) were found to be present in the galagan network (43), and these were also down-regulated in JAL (Fig. 7*D*). Among the up-regulated transcription factors the downstream targets of only two, Rv2989 and Rv3597c (Lsr2), were a part of the group of 257 proteins. Among the eight downstream targets of Rv2989, seven were found to be significantly up-regulated in JAL although one showed relatively higher expression compared with Rv. Interestingly, in the case of Lsr2 we observed a mixed pattern. At least 38 of the 601 targets of Lsr2 were either up- or down-regulated in JAL. Lsr2 is known to be a transcriptional repressor, and this mixed expression pattern observed signifies the interplay of complex regulatory mechanisms. The overall expression patterns of these transcription factors and their corresponding targets are consistent with previous reports (45–48).

##### Distinct Variations in Expression of Esx Contributes to the Survival Fitness of JAL

**FIGURE 8.**
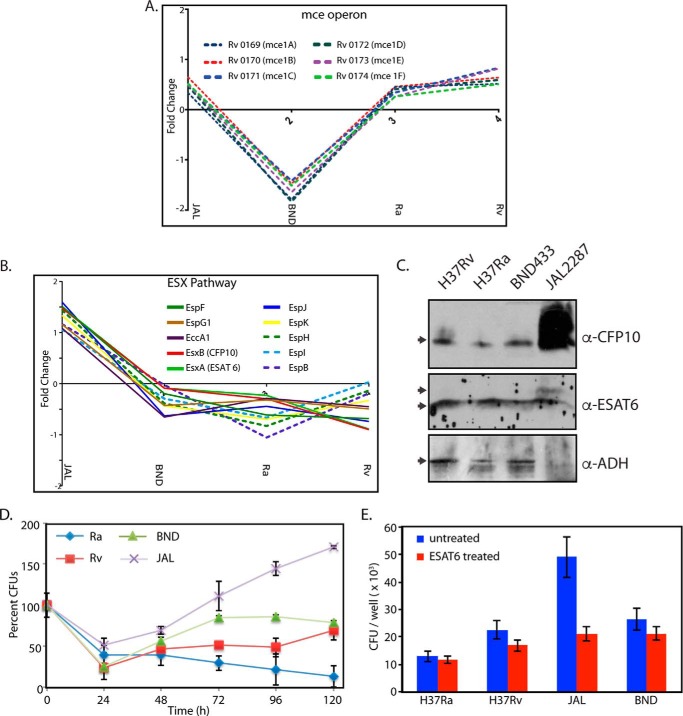
**Protein levels of Esx and Mce proteins vary among the strains.** *A,* profile plots showing the fold changes between different *M. tuberculosis* strains for the Mce proteins. *B,* profile plots showing the fold changes of different ESX pathway proteins (among the 257 proteins) between the four mycobacterial strains. *C,* culture filtrate fraction for each strain was estimated with the help of BCA assay kit. 15 μg of culture filtrates were resolved on SDS-PAGE, transferred to nitrocellulose membrane, and probed separately with α-CFP10, α-ESAT6, and α-ADH antibodies. *D,* THP1 cells differentiated with PMA were infected with Rv, Ra, BND, and JAL strains, and CFUs were enumerated at different time points post-infection. The CFUs at different time points for each strain were calculated with respect to the CFUs obtained at 0 h, which was normalized to 100. The experiment was performed in triplicate. and *error bars* represent S.E. *E,* uptake compromised with ESAT-6 antibody treatment. *M. tuberculosis* cultures were treated with ESAT-6 antibody (ESAT 6+) for 45 min at room temperature. Post-treatment, the cultures were washed and processed for infecting PMA-differentiated THP-1 cells at a multiplicity of infection of 10:1. As a control, untreated *M. tuberculosis* cultures were used. After 6 h post-infection, the cells were lysed and plated for obtaining CFU. The data represent average of values from three separate experiments.

Analysis of the data presented in Figs. 4 and 5 suggested lower expression of the entire *mce1* operon consisting of genes involved in the regulation of lipid metabolism in *M. tuberculosis* in the BND strain (Fig. 8*A*). This feature distinguished BND from the other three strains. Analysis of the 257 ANOVA-significant proteins showed that 10 proteins corresponding to the Esx family are up-regulated in JAL compared with the other three strains (Figs. 5, *cluster 1,* and 8*B*). Esx proteins are known to be critical for virulence, and their higher expression could be linked to the higher virulence of the JAL strain. Because the analysis was performed with the whole cell lysates, we sought to determine the levels of CFP10, ESAT6, and ADH in the culture filtrate fractions. As was the case with whole cell lysates (Fig. 4, *A* and *B*), ADH levels were observed to be lower in the culture filtrate fraction of JAL compared with the other strains (Fig. 8*C*). In agreement with the analysis, the levels of CFP10 in the culture filtrate fraction of JAL were found to be significantly higher (Fig. 8, *B* and *C*). However, in the case of ESAT6, an additional band migrating above the ESAT6 band was observed in the JAL lane (Fig. 8*C*), which could be due to some form of post-translational modification. To confirm the identity of the additional band in a separate experiment, we aligned the membrane with the Western blot, and both the bands were excised separately from the membrane. Mass spectrometry analysis of the tryptic peptides obtained from the membrane identified ESAT6 as the major protein in both the bands (60% coverage; five peptides were identified in each case), thus confirming the identity (data not shown). Consistent with the analysis (Fig. 8*B*), combined intensities of both the bands of ESAT6 in the JAL sample was higher compared with the other strains. Next, we sought to analyze whether the elevated levels of Esx proteins in JAL provide any survival advantage. Toward this, we performed *ex vivo* infections (THP1 cells) with all the strains and examined their survival post-infection. As expected, the Ra strain showed compromised survival. Although BND showed marginally compromised survival at the 24-h time point, the overall trend of survival in the differentiated profile was very similar to Rv. In contrast, the JAL strain seems to have considerable advantage in the host at every time point (Fig. 8*D*). Consistent with these results, we observed higher bacillary count, even at 6 h post-infection, in comparison with the other three strains in THP-1 cells infected with JAL strain (Fig. 8*E*). If the higher bacillary count observed in JAL were to be due to higher levels of secretory proteins such as ESAT-6, then decreasing ESAT-6 levels could be expected to compromise the bacillary count. Although pre-incubation of the bacteria with ESAT-6 antibody did not significantly alter the bacillary load in other strains, we observed an ∼2.5-fold decrease in the bacillary load in the JAL strain (Fig. 8*E*).

##### Possible Mechanisms for Drug Resistance

Drug resistance in *M. tuberculosis* can arise either due to modifications in the permeability barrier, up-regulation in the expression of efflux pump proteins, or genetic alterations of the target site in the target proteins (49). BND strain is resistant to streptomycin, and JAL strain is resistant to streptomycin, isoniazid, and rifampicin (36). To determine the relevance of known efflux pump proteins in the drug resistance, we looked at the 257 ANOVA-significant protein groups for their presence. With the exception of Rv1410c (50), a known drug efflux pump protein, and Rv2564, a hypothetical protein with ABC-like cassette, we did not find any other proteins with a transporter function. Both these proteins are down-regulated in JAL2287 but mostly unaffected in the other three strains thus omitting the possibility of their involvement.

To gain further insights into the possible mechanisms for drug resistance phenotypes, we mined the literature for candidates that have been up- or down-regulated in drug-resistant strains. We limited our analysis to proteins that are associated with streptomycin, isoniazid, and rifampicin resistance among the 257 ANOVA-significant protein groups. Unfortunately, the protein expression patterns for the streptomycin resistance phenotype of the BND strain did not comply with the existing literature (Table 1). Thus, we speculate that in case of the BND strain, the resistance could be due to the genetic alterations or modifications in the permeability barrier. The data (Table 1) could be broadly divided into two groups. In the first group, the previous observations in terms of regulation of a candidate protein were contrary to the protein levels observed in the multidrug-resistant JAL strain. These include the ABC transporter/efflux pump proteins, Rv3028 and Rv2933 (23, 50, 51). Rv2933 was identified to be up-regulated in a proteomic analysis of wild type and rifampicin-resistant Beijing clinical strains (52). Although we noticed up-regulation of Rv2933 in JAL and BND compared with Rv strain, its levels were much higher in rifampicin-sensitive Ra strain (Table 1). In the second group, the data from the literature were in agreement with the protein levels observed in JAL strain (Table 1). The quantitative proteomic analysis streptomycin-resistant isolates of *M. tuberculosis* have shown decreased expression of Rv0824 and Rv3133 (DevR) (53). Down-regulation of DevR has also been implicated in the hypervirulence of *M. tuberculosis* resulting in early death of SCID mice (54). Interestingly, we observed significant down-regulation of Rv0824 and DevR in the JAL strain, which may explain streptomycin resistance as well as hypervirulence phenotypes. Proteomic analysis of streptomycin- or isoniazid-resistant strains has revealed up-regulation of a number of proteins (23, 51). Few among these proteins such as Rv2145c, Rv1240, Rv2971, Rv0560c, and Rv1446c were significantly up-regulated in the JAL strain (Table 1). Isoniazid was shown to induce the expression of propionyl-CoA carboxylase β-chain 6 (Rv2247) (15). Similarly, overexpression of isoniazid-induced protein (Rv0341-IniB) led to prolonged survival of an otherwise sensitive strain of *M. tuberculosis* even at an inhibitory concentration of isoniazid (55). Both of these proteins were found to be up-regulated in JAL compared with the other strains. Taken together, the isoniazid and streptomycin resistance phenotype of JAL strain could be due to the overexpression of multiple candidate proteins (Table 1). However, we have not found any such correlation for rifampicin resistance phenotype of JAL, which could either be due to altered permeability or genetic alterations. Thus we speculate that the multidrug resistance phenotype of JAL could be due to a combination of overexpression of proteins implicated in drug resistance (Table 1), genetic alterations, and/or altered permeability.

**TABLE 1:** **Candidate proteins that are either up- or down-regulated in the drug-resistant strains** Analysis was limited to streptomycin, isoniazid, and rifampicin resistance among the 257 ANOVA-significant protein groups. Details of gene identification number, protein name, the median values observed for each strain, and the plausible function have been provided.

Gene	Protein names	JAL	BND	Ra	Rv	Function	Ref.
**Candidate proteins with transport function**							
Rv2564	Uncharacterized ABC transporter ATP-binding protein	−1.45907	−0.0728709	0.915215	0.62991	Predicted to be a glutamine ABC transporter	
Rv1410c	MFS-type drug efflux transporter P55	−1.16128	0.368715	0.82969	0.121877	Multidrug efflux pump protein; overexpression in BCG conferred streptomycin and tetracycline resistance	50

**Candidates for which the regulation of expression was contrary to the protein levels observed in JAL strain**							
Rv3028c	Electron transfer flavoprotein subunit α	−1.03707	0.646106	0.392637	0.0287605	Overexpressed in streptomycin-and isoniazid-resistant clinical isolates	23, 51
Rv2933	Phthiocerol synthesis polyketide synthase type I PpsC	0.347323	0.43145	1.01702	−1.34932	Up-regulated in rifampicin-resistant-*rpoB* mutant *Mtb*	52

**Candidates for which the data from the literature was in agreement with the protein levels observed in JAL strain**							
Rv0824c	Acyl-[acyl-carrier-protein] desaturase desA1	−1.50968	0.689537	0.492385	0.402516	Down-regulated in streptomycin- resistant *Mtb*	53
Rv3133c	Transcriptional regulatory protein DevR (DR)	−1.48441	0.568373	0.255834	0.572789	Downregulated in streptomycin resistant *Mtb*	53, 54
Rv2145c	Cell wall synthesis protein-Wag31	0.90589	−0.100451	−1.19427	0.280966	Overexpressed in streptomycin- and isoniazid-resistant clinical isolates	23, 51
Rv0341	Isoniazid-induced protein-IniB	1.1082	−1.14845	−0.584435	0.231656	Strains overexpressing IniB survive longer at inhibitory concentration of isoniazid	55
Rv1240	Malate dehydrogenase	1.23081	−0.333817	−0.463767	−0.618897	Overexpressed in streptomycin-resistant clinical isolates	51
Rv0560c	Uncharacterized protein Rv0560c	1.38842	−0.254523	−0.293821	−0.273248	Overexpressed in streptomycin-resistant clinical isolates	51
Rv2971	Uncharacterized oxidoreductase Rv2971	1.568	0.00266458	−0.633307	−0.799756	Overexpressed in streptomycin- and isoniazid-resistant clinical isolates	23, 51
Rv2247	Propionyl-CoA carboxylase β-chain 6	1.40693	−0.539375	−0.877811	0.0798006	Expression induced in isoniazid-dependent manner	15
Rv1446c	OXPP cycle protein OpcA	1.52307	−0.283431	−1.04449	−0.240305	Overexpressed in isoniazid-resistant clinical isolates	23

## Discussion

Here, we report a comprehensive analysis of the proteomes of four different strains of *Mycobacterium* that are avirulent laboratory strain, virulent laboratory strain, single drug-resistant clinical isolate, and multidrug-resistant clinical isolate, respectively. The depth of coverage of ∼54% was achieved with a fairly simple proteomic workflow in a single run that did not involve labeling or pre-fractionation of peptides. Correlation analysis of the four biological replicates of each strain showed *R*-values greater than 0.82, confirming their suitability for further statistical analysis. Utilization of label-free quantification methodology to discover strain-specific proteomic differences revealed that 257 protein groups were differentially expressed among the four strains.

K-means cluster analysis of the 257 proteins suggested seven distinct expression patterns. Cluster 1 contained proteins such as RegX1 (Rv0491), a constituent of the two-component regulatory system, as well as Rv2971 (probable oxidoreductase) that are highly expressed in isoniazid-resistant *M. tuberculosis* strains (23). Other interesting candidates were HtrA, serine protease (Rv1223; previously reported to be a major virulence factor of *Streptococcus pneumoniae* in an *in vivo* pneumonia model (56–58)), and KasB (Rv2246; causes subclinical latent tuberculosis in immunocompetent mice (59–61)). Many of the proteins in the clusters 2 and 5 are related to ribosome biogenesis and energy generation. Rv1446 (OpcA) and Rv2145c (Wag31; plays a role in peptidoglycan synthesis and regulating cell shape and oxidative response) that are expressed in higher levels in isoniazid-resistant strains are part of cluster 2 (23, 62, 63). The higher expression of these proteins in JAL compared with the other strains is consistent with its multidrug resistance and implicates a role for these proteins in mediating cell survival under stress.

Interestingly, virulence-related proteins, such as Rv2780 (l-alanine dehydrogenase), Rv0126 (TreS), Rv2299c (HtpG), two-component system proteins like Rv0042c, Rv903c (PrrA), and Rv3133c (DevR), were present at lower levels in JAL. l-Alanine dehydrogenase was the first antigen reported to be absent in the *M. bovis* BCG (vaccine strain) (18, 64). Two component systems are studied well in multiple organisms and are known to coordinate gene expression under different environmental conditions (65, 66). Most of the target genes of DosR were part of clusters 4 and 6. Down-regulation of two-component signaling in JAL might suggest a role for two-component signaling in drug resistance (45). Although the identified sub-clusters only partially represent the global regulatory perturbations specific to any strain, a significantly higher flux through the regulatory network in case of JAL as compared with other three strains is certainly suggested by the higher magnitude of protein regulation in this strain.

Rv3597c (Lsr2) is considered to be a global transcriptional regulator that represses a large number of virulence-related genes (46–48). A recent report has demonstrated the crucial role of Lsr2 in hypoxia adaptation as well as persistent infection inside the host (67). In this study most of the Lsr2 targets were present in clusters 1 and 2 and a few of them in cluster 7. Our results show the up-regulation of Lsr2 and Rv2989 and down-regulation of DosR in JAL as compared with the other strains. A corresponding modulation of expression of a number of their targets was also observed. Although it is tempting to directly correlate the expression of the target proteins, those of the transcription factors that regulate them, one cannot rule out additional tiers of regulation.

Cluster 3 contained 17 proteins, which showed low expression in BND and high expression in all the other three strains. Interestingly, proteins expressed by the *mce1* operon were found to be down-regulated in the BND strain. The *M. tuberculosis* genome contains four *mce* operons, thought to be important for replication in mice (68, 69). The deletion of the *mce1* operon from *M. tuberculosis* has been shown earlier to impact the lipid profiles and uptake of palmitic acid and accumulation of more mycolic acids in comparison with the wild type (40). Cholesterol import and metabolism are dependent on the proteins encoded by the *mce4* operon (70). We performed experiments to determine whether this difference would be reflected in the growth of the strains in different carbon source. Although we observed slower growth of BND compared with the other strains in the early stages of growth, the differences in the growth were nullified when following the growth for a longer duration (data not shown). One possible explanation for this phenotype is that, although the *mce1* operon may be playing a role in adaptation in the early stages of growth in the BND strain, alternative pathways (other *mce* operons) may be compensating for the down-regulation of the *mce1* operon in the later stages.

The difference in the virulence between H37Ra and H37Rv has been largely attributed to a point mutation in the two-component system protein PhoP, which is a transcription factor known to regulate a variety of genes, including the genes in the *esx1* locus (71, 72). Comparison of secreted proteome from H37Ra and H37Rv using two-dimensional gel followed by mass spectrometry, showed several proteins with putative ESAT-6 like function to be present at higher levels in H37Rv compared with H37Ra (73). However, Frigui *et al.* (72) showed complete loss of ESAT-6 secretion in the attenuated strain H37Ra. In yet another study, Malen *et al.* (74) performed membrane proteomics of H37Ra and H37Rv and did not find lower expression of ESAT-6 in the membrane fraction. They did observe decreased expression of several of the PhoP target genes in their analysis. Interestingly, we did not observe significant differences in the cellular or secreted levels of ESAT-6 in H37Ra compared with H37Rv. We followed the Malen *et al.* (74) strategy and looked at the protein expression levels of PhoP direct targets. We could find 16 of those genes in our data set, most of which did not show any significant regulation in either H37Ra or H37Rv except for two of them (Rv2145c and Rv3881c (EspB)), which showed significant down-regulation in case or H37Ra. EspB, a substrate of ESX-1, is known to be required for virulence and growth in macrophages (75).

The protein members of the ESX-1 family are potent T-cell antigens that play a critical biological role in interactions with host cells and are thought to be important for virulence and pathogenesis (29, 76, 77). Because the ESX-1 machinery is up-regulated in JAL compared with the other three strains, one would expect higher levels of ESAT6 and CFP10 proteins in the culture filtrate fraction. Consistent with the hypothesis that JAL may have higher ESX-1 activity, we observed higher levels of ESAT6 and CFP10 in the culture filtrate fraction (Fig. 8, *B* and *C*). In agreement with their proposed role in virulence and pathogenesis, JAL strain survival in PMA-differentiated THP1 cells was higher than the other strains (Fig. 8*D*). JAL strain seems to have the advantage at every time point tested, indicating that the higher ESX-1 activity may be providing survival advantage to JAL compared with the other strains. Furthermore, the survival advantage observed seems be dependent on ESAT-6 levels as pre-incubation with the ESAT-6 antibody significantly compromised the intracellular bacterial load (Fig. 8*E*). Metabolic network analysis (Fig. 6, *B* and *C,* and supplemental Table 9) revealed that even though a number of proteins associated with lipid metabolism are down-regulated in JAL, there was at least one protein-associated β-oxidation with each of the four reactions in the pathway that was up-regulated (see Fig. 6*C*). The fact that JAL has alternative pathways for lipid metabolism and has an efficient ESX-1 secretion system would explain the survival advantage compared with other strains. Taken together, the data presented here illustrate the potency of the proteomic approach to select candidates that are differentially expressed in clinical strains, the relevance of which can be characterized further using cell and molecular biology tools.

## Author Contributions

G. D. J., S. K., and V. K. N. conceived and coordinated the study. G. D. J. and S. K. performed the experiments and acquired and analyzed the data. D. A. performed growth curve and Western blotting analysis. S. V. J. and N. J. performed and analyzed the experiment shown in Fig. 8. K. V. S. R. analyzed the experiments in Fig. 8 and helped in overall analysis. H. K. and D. K. analyzed the large scale proteomic data. D. K. performed and coordinated the K-cluster and PCA analyses. A. S. and L. K. K. performed metabolic network analysis. G. D. J., S. K., A. S., D. K., and V. K. N. wrote the paper. All authors reviewed the results and approved the final version of the manuscript.

## Supplementary Material

Supplemental Data

## Footnotes

3 The abbreviations used are:

iBAQ: intensity-based absolute quantification
ANOVA: analysis of variance
PCA: principal component analysis
ADH: alanine dehydrogenase
LFQ: label-free quantification
PC: principal component
ABC: ATP-binding cassette
PMA: phorbol 12-myristate 13-acetate.
