# Supplementary material for: Comparative Proteomic Analyses of Avirulent, Virulent, and Clinical Strains of Mycobacterium tuberculosis Identify Strain-specific Patterns
Source: J Biol Chem. 2016 May 5;291(27):14257–73. doi: 10.1074/jbc.M115.666123 (PMC4933181; doi:10.1074/jbc.M115.666123)
Supplement: Supplemental Data [file supp_291_27_14257__index.html]

Comparative proteomic analyses of avirulent, virulent and clinical strains of Mycobacterium tuberculosis identifies strain-specific patterns — Comparative Proteomic Analyses of Avirulent, Virulent, and Clinical Strains of Mycobacterium tuberculosis Identify Strain-specific Patterns — Strain-specific Protein Expression Profiles in M. tuberculosis — Supplemental Data 

# Comparative Proteomic Analyses of Avirulent, Virulent, and Clinical Strains of *Mycobacterium tuberculosis* Identify Strain-specific Patterns

## Supplemental Data

- Supplemental Table 1 (.xlsx, 1.4 MB) - The complete protein identification list for all four strains
- Supplemental Table 2 (.xlsx, 3.3 MB) - The total protein group list for all four strains with coverage details
- Supplemental Table 3 (.xlsx, 6.7 MB) - The total peptide sequences detected among the four strains with identification details
- Supplemental Table 4 (.pdf, 680 KB) - Comparative table showing the strain specific proteins in four biological replicates
- Supplemental Table 5 (.xlsx, 164 KB) - proteins identified among all the four strains were ranked based on combined normalized log2 transformed iBAQ values
- Supplemental Table 6 (.xlsx, 232 KB) - The complete list of proteins that passed ANOVA significance test
- Supplemental Table 7 (.xlsx, 110 KB) - Summary statistics of 257 ANOVA significant proteins. Mean, median and standard deviation among the replicates of each strain are represented.
- 86 (.xlsx, 34 KB) - The complete protein identification list based on K- means clustering
- Supplemental Table 9 (.xlsx, 39 KB) - Subsystem based classification of the differentialy expressed proteins in the four MTB strains.
