# Supplementary material for: Comparative Proteomic Analyses of Avirulent, Virulent, and Clinical Strains of Mycobacterium tuberculosis Identify Strain-specific Patterns
Source: J Biol Chem. 2016 May 5;291(27):14257–73. doi: 10.1074/jbc.M115.666123 (PMC4933181; doi:10.1074/jbc.M115.666123)
Supplement: Supplemental Data [file 10.1074_M115.666123_jbc.M115.666123-4.pdf]

Supplementary Table4: Comparative table showing strain specific proteins based on four biological replicates

highlighted cells indicates the strain specific proteins present in two or more bio replicates

Biological replicates and number of peptides identified																			
**Rv1**	**Rv2**	**Rv3**	**Rv4**	**Ra1**	**Ra2**	**Ra3**	**Ra4**	**BND1**	**BND2**	**BND3**	**BND4**	**JAL1**	**JAL2**	**JAL3**	**JAL4**	**Protein IDs**	**Uniprot names**	**Gene name**	**Protein name**
1	NA	NA	NA	NA	NA	NA	NA	NA	NA	NA	NA	NA	NA	NA	NA	L0TES5	L0TES5	Rv3251c	Rubredoxin
NA	NA	1	NA	NA	NA	NA	NA	NA	NA	NA	NA	NA	NA	NA	NA	L7N4G1	L7N4G1	Rv0348	Possible transcriptional regulatory protein
1	1	NA	NA	NA	NA	NA	NA	NA	NA	NA	NA	NA	NA	NA	NA	L7N5E4	L7N5E4	Rv1393c	Monooxygenase, flavin-binding family
1	NA	3	1	NA	NA	NA	NA	NA	NA	NA	NA	NA	NA	NA	NA	O53624	O53624	Rv0079	Uncharacterized protein Rv0079/MT0086
1	NA	NA	NA	NA	NA	NA	NA	NA	NA	NA	NA	NA	NA	NA	NA	O53768	O53768	Rv0571c	phosphoribyl transferase Rv0571c/MT0597
1	NA	NA	NA	NA	NA	NA	NA	NA	NA	NA	NA	NA	NA	NA	NA	P0A562	P0A562	Rv2364c	GTPase Era
NA	1	NA	NA	NA	NA	NA	NA	NA	NA	NA	NA	NA	NA	NA	NA	P0A660	P0A660	Rv1850	Urease subunit alpha
NA	NA	NA	1	NA	NA	NA	NA	NA	NA	NA	NA	NA	NA	NA	NA	P0A668	P0A668	Rv2875	Immunogenic protein MPT70
1	NA	NA	NA	NA	NA	NA	NA	NA	NA	NA	NA	NA	NA	NA	NA	P64705	P64705	Rv0485	Uncharacterized protein Rv0485/MT0503
1	NA	NA	NA	NA	NA	NA	NA	NA	NA	NA	NA	NA	NA	NA	NA	P64741	P64741	Rv0887c	Uncharacterized protein Rv0887c/MT0910
NA	NA	1	NA	NA	NA	NA	NA	NA	NA	NA	NA	NA	NA	NA	NA	P64773	P64773	Rv0960	ribonuclease VapC9
1	1	NA	NA	NA	NA	NA	NA	NA	NA	NA	NA	NA	NA	NA	NA	P95027	P95027	Rv2526	antitoxin VapB17
NA	NA	2	NA	NA	NA	NA	NA	NA	NA	NA	NA	NA	NA	NA	NA	P96263	P96263	Rv0417	Thiazole synthase
NA	NA	NA	NA	1	NA	NA	NA	NA	NA	NA	NA	NA	NA	NA	NA	L0T6B1	L0T6B1	Rv0317c	glycerophphoryl diester phphodiesterase GlpQ2
NA	NA	NA	NA	NA	NA	1	NA	NA	NA	NA	NA	NA	NA	NA	NA	L0T7I3	L0T7I3	Rv1730c	penicillin-binding protein
NA	NA	NA	NA	1	NA	1	NA	NA	NA	NA	NA	NA	NA	NA	NA	L0T826	L0T826	Rv0974c	acetyl-/propionyl-CoA carboxylase (Beta subunit) AccD2
NA	NA	NA	NA	NA	NA	NA	2	NA	NA	NA	NA	NA	NA	NA	NA	L7N4Z6	L7N4Z6	Rv1187	Delta-1-pyrroline-5-carboxylate dehydrogenase
NA	NA	NA	NA	NA	NA	NA	1	NA	NA	NA	NA	NA	NA	NA	NA	L7N554	L7N554	Rv0067c	Possible transcriptional regulatory protein (Psibly TetR-family)
NA	NA	NA	NA	1	NA	NA	NA	NA	NA	NA	NA	NA	NA	NA	NA	L7N5K3	L7N5K3	Rv0186	Beta-glucidase,
NA	NA	NA	NA	1	NA	NA	NA	NA	NA	NA	NA	NA	NA	NA	NA	O06197	O06197	Rv2617c	transmembrane protein
NA	NA	NA	NA	1	NA	NA	NA	NA	NA	NA	NA	NA	NA	NA	NA	P63911	P63911	Rv2637	Uncharacterized membrane protein Rv2637/MT2715
NA	NA	NA	NA	NA	1	NA	NA	NA	NA	NA	NA	NA	NA	NA	NA	P71880	P71880	Rv2332	malate oxidoreductase [NAD]
NA	NA	NA	NA	1	1	NA	1	NA	NA	NA	NA	NA	NA	NA	NA	P96843	P96843	Rv3561	fatty-acid-CoA ligase FadD3
NA	NA	NA	NA	1	NA	NA	NA	NA	NA	NA	NA	NA	NA	NA	NA	Q10528	Q10528	Rv2250c	Uncharacterized HTH-type transcriptional regulator
NA	NA	NA	NA	NA	1	NA	NA	NA	NA	NA	NA	NA	NA	NA	NA	Q10859	Q10859	Rv1998c	Uncharacterized protein Rv1998c/MT2054
NA	NA	NA	NA	NA	NA	NA	NA	NA	NA	NA	1	NA	NA	NA	NA	L7N4A9	L7N4A9	Rv1174c	Low molecular weight T-cell antigen TB8.4
NA	NA	NA	NA	NA	NA	NA	NA	NA	1	NA	NA	NA	NA	NA	NA	L7N4Z9	L7N4Z9	Rv1709	segregation and condensation protein ScpA
NA	NA	NA	NA	NA	NA	NA	NA	NA	NA	NA	1	NA	NA	NA	NA	L7N574	L7N574	Rv2375	Uncharacterized protein
NA	NA	NA	NA	NA	NA	NA	NA	1	3	NA	NA	NA	NA	NA	NA	L7N5Q9	L7N5Q9	Rv0123	DNA-binding protein, CopG family
NA	NA	NA	NA	NA	NA	NA	NA	NA	1	NA	NA	NA	NA	NA	NA	O53301	O53301	Rv3084	acetyl-hydrolase LipR
NA	NA	NA	NA	NA	NA	NA	NA	NA	NA	1	1	NA	NA	NA	NA	O53788	O53788	Rv0680c	conserved transmembrane protein
NA	NA	NA	NA	NA	NA	NA	NA	NA	1	NA	NA	NA	NA	NA	NA	P67731	P67731	Rv1994c	HTH-type transcriptional regulator CmtR
NA	NA	NA	NA	NA	NA	NA	NA	NA	1	NA	NA	NA	NA	NA	NA	P69419	P69419	Rv2922.1c Rv2922A	Acylphphatase
NA	NA	NA	NA	NA	NA	NA	NA	1	NA	NA	NA	NA	NA	NA	NA	P71821	P71821	Rv0765c	Oxidoreductase, short-chain dehydrogenase/reductase family
NA	NA	NA	NA	NA	NA	NA	NA	NA	NA	NA	1	NA	NA	NA	NA	P71825	P71825	Rv0770	Uncharacterized oxidoreductase Rv0770/MT0794
NA	NA	NA	NA	NA	NA	NA	NA	NA	1	1	NA	NA	NA	NA	NA	P95024	P95024	Rv2529	Uncharacterized protein
NA	NA	NA	NA	NA	NA	NA	NA	1	NA	NA	2	NA	NA	NA	NA	P95110	P95110	Rv2985	8-oxo-dGTP diphphatase 1
NA	NA	NA	NA	NA	NA	NA	NA	NA	NA	NA	1	NA	NA	NA	NA	P96887	P96887	Rv3282	Maf-like protein Rv3282/MT3381
NA	NA	NA	NA	NA	NA	NA	NA	NA	NA	NA	NA	1	NA	1	1	L0T8E5	L0T8E5	Rv2024c	Uncharacterized protein
NA	NA	NA	NA	NA	NA	NA	NA	NA	NA	NA	NA	1	1	1	1	L0TA18	L0TA18	Rv1644	23S rRNA methyltransferase TsnR
NA	NA	NA	NA	NA	NA	NA	NA	NA	NA	NA	NA	1	1	NA	1	L0TAM4	L0TAM4	Rv1864c	Conserved protein
NA	NA	NA	NA	NA	NA	NA	NA	NA	NA	NA	NA	NA	NA	1	1	L7N4U9	L7N4U9	Rv1615	membrane protein
NA	NA	NA	NA	NA	NA	NA	NA	NA	NA	NA	NA	NA	NA	1	NA	L7N5G7	L7N5G7	Rv1976c	Uncharacterized protein
NA	NA	NA	NA	NA	NA	NA	NA	NA	NA	NA	NA	NA	NA	1	1	O06279	O06279	Rv3603c	Conserved hypothetical alanine and leucine rich protein
NA	NA	NA	NA	NA	NA	NA	NA	NA	NA	NA	NA	NA	NA	1	1	O33302	O33302	Rv2760c	antitoxin VapB42
NA	NA	NA	NA	NA	NA	NA	NA	NA	NA	NA	NA	NA	1	1	1	O53168	O53168	Rv1477	Peptidoglycan endopeptidase RipA
NA	NA	NA	NA	NA	NA	NA	NA	NA	NA	NA	NA	2	2	2	2	O53374	O53374	Rv3322c	methyltransferase
NA	NA	NA	NA	NA	NA	NA	NA	NA	NA	NA	NA	1	NA	1	1	O53610	O53610	Rv0065	ribonuclease VapC1
NA	NA	NA	NA	NA	NA	NA	NA	NA	NA	NA	NA	1	NA	NA	1	O86328	O86328	Rv2421c	nicotinate-nucleotide adenylyltransferase
NA	NA	NA	NA	NA	NA	NA	NA	NA	NA	NA	NA	NA	NA	1	1	P0A676	P0A676	Rv1848	Urease subunit gamma
NA	NA	NA	NA	NA	NA	NA	NA	NA	NA	NA	NA	1	1	1	1	P0CW29	P0CW29	Rv0064A	antitoxin VapB1
NA	NA	NA	NA	NA	NA	NA	NA	NA	NA	NA	NA	2	2	2	4	P65392	P65392	Rv3324c	Cyclic pyranopterin monophphate synthase accessory protein 3
NA	NA	NA	NA	NA	NA	NA	NA	NA	NA	NA	NA	NA	1	NA	NA	P67134	P67134	Rv2367c	Endoribonuclease YbeY
NA	NA	NA	NA	NA	NA	NA	NA	NA	NA	NA	NA	NA	NA	NA	1	P71650	P71650	Rv2801c	mRNA interferase MazF9
NA	NA	NA	NA	NA	NA	NA	NA	NA	NA	NA	NA	1	1	NA	1	P71835	P71835	Rv0781	protease II PtrBa [first part] (Oligopeptidase B)
NA	NA	NA	NA	NA	NA	NA	NA	NA	NA	NA	NA	NA	NA	1	NA	P95003	P95003	Rv2550c	Antitoxin VapB20
NA	NA	NA	NA	NA	NA	NA	NA	NA	NA	NA	NA	NA	NA	1	1	P95106	P95106	Rv3053c	Glutaredoxin NrdH,
NA	NA	NA	NA	NA	NA	NA	NA	NA	NA	NA	NA	1	2	1	1	Q50687	Q50687	Rv2277c	Uncharacterized protein Rv2277c/MT2337
NA	NA	NA	NA	NA	NA	NA	NA	NA	NA	NA	NA	2	2	1	1	Q50737	Q50737	Rv2561/Rv2562	Uncharacterized protein Rv2561/Rv2562/MT2638
NA	NA	NA	NA	NA	NA	NA	NA	NA	NA	NA	NA	NA	NA	1	1	Q8VJ59	Q8VJ59	Rv3190A	Conserved protein
